# MobChain: Three-Way Collusion Resistance in Witness-Oriented Location Proof Systems Using Distributed Consensus

**DOI:** 10.3390/s21155096

**Published:** 2021-07-28

**Authors:** Faheem Zafar, Abid Khan, Saif Ur Rehman Malik, Mansoor Ahmed, Carsten Maple, Adeel Anjum

**Affiliations:** 1 Department of Computer Science, COMSATS University Islamabad (CUI), Islamabad 42000, Pakistan; faheemiiui@gmail.com (F.Z.); mansoor@comsats.edu.pk (M.A.); adeel.anjum@comsats.edu.pk (A.A.); 2 Department of Computer Science, Aberystwyth University, Aberystwyth SY23 3DB, UK; abk15@aber.ac.uk; 3 Cybernetica AS, 12618 Tallinn, Estonia; saif.rehmanmalik@cyber.ee; 4 Secure Cyber Systems Research Group, WMG, University of Warwick, Coventry CV4 7AL, UK

**Keywords:** location-based services (LBS), location proof, location provenance, localization, blockchain

## Abstract

Smart devices have accentuated the importance of geolocation information. Geolocation identification using smart devices has paved the path for incentive-based location-based services (LBS). However, a user’s full control over a smart device can allow tampering of the location proof. Witness-oriented location proof systems (LPS) have emerged to resist the generation of false proofs and mitigate collusion attacks. However, witness-oriented LPS are still susceptible to three-way collusion attacks (involving the user, location authority, and the witness). To overcome the threat of three-way collusion in existing schemes, we introduce a decentralized consensus protocol called MobChain in this paper. In this scheme the selection of a witness and location authority is achieved through a distributed consensus of nodes in an underlying P2P network that establishes a private blockchain. The persistent provenance data over the blockchain provides strong security guarantees; as a result, the forging and manipulation of location becomes impractical. MobChain provides secure location provenance architecture, relying on decentralized decision making for the selection of participants of the protocol thereby addressing the three-way collusion problem. Our prototype implementation and comparison with the state-of-the-art solutions show that MobChain is computationally efficient and highly available while improving the security of LPS.

## 1. Introduction

Location-based services (LBS) have reformed business models by offering services based on the current geographical location of users. The importance of smart devices to provide a person’s accurate geographical location is heightened when accessing location-based services. Consumer-based applications such as locator services (nearest restaurants, stores, ATM, etc.), location-based content (games, news, weather updates, etc.), location-based social networks (LBSN), and vehicle route guidance have been made available through smart devices [1]. Beyond consumer-based services in businesses such as courier services and pharmaceutical distributors, location information can improve business operations in the field using smart devices. The real challenge is to prove that the person was physically present at the reported location and time. Location data provided by the individuals themselves may be falsified and so should not be considered trusted. The user may cheat to earn the incentives or to hide noncompliance to job responsibilities. With the advent of the location proof system (LPS), it is now possible to achieve trustworthy proof of user physical presence at a location using smart devices. LPS aids in the generation of a location proof (LP) [2], which is a digitally signed asset certifying the user presence at a geographical location at a specific time. A LP contains the user identity, location coordinates, and a timestamp, all of which is verifiable in a secure manner. To better understand the importance of the LPS, the following are a few real-world examples of its application: (a) A utility store might offer special discounts on a customer’s loyalty. To claim the discounts, customers may use their smart devices to get proof of every visit from the store and present them in the future as the history of the visit; (b) A special task force assigned to a critical mission in the enemy area may be asked to keep a copy of their location traces such that the commanding center may perform post-mission analysis of navigation; (c) Transportation incentives may encourage travelling along environment-friendly routes; (d) A courier service delivering mobile bills may ask the employees to get location proofs to ensure that they have delivered the bills to the customer’s location, ensuring the quality of the operation; (e) Pharmaceutical companies can utilize the location proofs of medical representatives and salespersons to ensure that they are visiting hospitals and doctors [3]; (f) Construction companies can benefit by utilizing location proofs to ensure that engineers visiting sites; and (g) Location-based access control [4].

To better grasp the idea of location proof systems, the following terminologies must be understood [5]:

- Prover: A prover is a user who visits a particular location at a specific instance of the time. The user can identify his current location using a smart device that wants to generate digital proof of his physical presence. Digital proof of the physical presence of the prover can be verified in the future.
- Location: A geographical region having defined area boundaries with designated location authorities.
- Location Authority (LA): A location authority is a designated stationery entity on a specific location. The LA assists in proof generation for a prover’s physical presence at a location in a secure manner.
- Location Proof Assertion/Endorsement: An assertion/endorsement of location proof is a digital statement issued by an entity involved in the process of proof generation. An assertion statement guarantees the validity of the process and information contained in a location proof.
- Witness: Witnesses are mobile entities with smart devices, which are temporarily present at a specific location. The witness is engaged by the system to endorse the location proof by attesting the physical presence of the prover co-located with LA.
- Location Provenance: A location provenance is a chain of location proofs tracking the travel history of a prover, preserving the chronological order of locations visited.
- Auditor: An auditor is a semi-trusted entity that validates the location proof of the prover. Furthermore, the auditor is capable of validating the travel path of the prover using location provenance.

LPS uses the “localization” concept by which a smart device can report its location relative to the co-located device or cellular tower or using a global positioning system (GPS) [6]. For localization, proof generation systems have been designed that utilize Bluetooth [7], GPS [8], Wi-Fi infrastructure [9], and infrared [10]. Other techniques such as distance-bounding protocols [11] and proximity [6,12], cellular tower triangulation, mobile device triangulation, IP address tracking [3] and audio-based positioning [10] have also been used to generate location proofs in the past. Initially, systems were designed using a centralized architecture with a trusted third party to validate the user’s location claims. However, centralized LPS suffer from a single point of failure which can result in performance issues in peak load times. Limitations of centralized LPS have been mitigated by introducing a distributed LPS architecture [9]. Distributed LPS started with a two-party protocol (involving the prover and location authority) and have gradually evolved to witness-oriented three-party (prover, location authority, witness) and multi-party (prover, location authority, multiple witnesses) protocols for a location-proof generation. However, distributed LPS are prone to collusion attacks. Many approaches have been used to mitigate collusion attacks using outlier detection techniques [7], entropy-based trust model along with localization using distance bound protocol [9], and witness-asserted location proofs [13]. Latterly, LPS have evolved to support location provenance. Location provenance chaining tracks the location history of the traveled path in a secure manner by keeping the chronological order of locations visited. The chronological order of location information is of great importance to increase the reliability of location proofs. Location provenance chaining can be also used to detect attacks such as backdating or future dating. Furthermore, a trust evaluation system can also be designed over a location provenance chain. For example, a trust evaluation mechanism can be based on spatio-temporal correlation, where the time difference and distance between two consecutive location proofs can aid in the determination of a false proof [14]. Consider a case where a user can successfully obtain location proof LP1 for location A at time instance T1 and LP2 for location B at a later time instance T2. Now, if T2–T1 is so short that the user cannot practically travel from A to B in this time, the existence of a false proof can be identified.

Nowadays, blockchain-based provenance schemes are increasing in popularity for resilience and security reasons [15]. Blockchains [16,17] are well known as immutable distributed ledgers. Contrary to a centralized database, a distributed ledger is a database where records are stored and confirmed by multiple participants through a distributed consensus mechanism. However, a single authority has control over the network of the distributed ledger, making it partially decentralized. Blockchain, on the other hand, is fully decentralized. Full decentralization means that no single entity controls the network of blockchain and data are stored over it through the distributed consensus of all nodes in the network. Therefore, blockchain can be considered to be an extension of a distributed ledger. Blockchain technology is a timestamped immutable chain of blocks chained together using a cryptographic mechanism. Furthermore, the blockchain consensus algorithm [18] ensures that all nodes have agreement on the state of the chain containing data blocks. A blockchain satisfies the following properties, which may or may not be satisfied by all distributed ledgers:

- Accountability: All participant nodes in blockchain (except thin nodes) are capable of validating the transactions in the block and can accept or reject blocks on verification. Therefore, the state of the chain gets verified by participating nodes along with every single transaction, providing strong accountability.
- Tamper-resistant: Blockchain’s decentralized mechanism of a block’s chain storage with the participation of a large number of nodes makes it irreversible and immutable. Therefore, blockchain data become tamper resistant over a period of time.
- Distributed data control: Blockchain removes the single point of failure by decentralization of data and its control and therefore provides high availability.
- No trusted third-party dependency: The blockchain consensus mechanism ensures the state of a chain without reliance on a trusted third-party.
- Non-repudiation: Transactions in blocks of blockchain are signed by public-key cryptography ensuring the authenticity of the data. The immutable and irreversible nature of the chain with cryptographic mechanism provides non-repudiation.

Furthermore, immutability and implicit record chaining in blockchain have facilitated its application in systems such as recording the chain of custody in supply-chain management systems [19,20], data provenance management in cloud storage [21], privacy-preserving smart contracts [22,23] and decentralized file storage [24,25]. However, the application of blockchain for location provenance management to record the chronological order of the travel history securely is currently gaining the attention of the research community. To the best of our knowledge, Giacomo et al. [26] proposed the first decentralized LPS using blockchain for P2P overlay schemes. They used blockchain for storing location provenance data as a method to resist backdating and future dating attacks. Later, decentralized LPS such as [27,28] were also proposed based on the P2P network of blockchain.

### 1.1. Requirements for Location Proof

For location proof to be trustable, the following are the minimum requirements in LPS [5,9]:

- Integrity: Any prover should not be able to generate a fake location proof. Any fake location proof must be detectable. The LA and witnesses should not be able to victimize the prover to provide a fake location proof.
- Non-transferability: Any prover should not be able to falsely claim ownership of any legitimate location proof generated for any other prover.
- Trustworthiness: Any self-reported location proof should not be trusted. Therefore, attestation of the location proofs is required from other entities (such as the location authority or a witness) in the system to make them trustable.
- Non-repudiation: To mitigate the scenario of a user denying a location visit, a location proof, once generated by a user, should not be able to be denied.

### 1.2. Requirements for Location Provenance Chain

The following are the requirements that must be satisfied by a location provenance system [5,13,29]:

- Chronological Order: The order of the location proofs recorded in the provenance chain must depict the same sequence as the locations visited by the user.
- Timestamping: In a distributed configuration, the clocks of the entities in a location-proof system can be different; the clock of the user’s smart device should not be trusted. Therefore, accurate timestamping is a requirement. It is a challenge to accurately record the chronological order of location proofs of user visits.
- Tamper Evident: The location provenance chain should be tamper-evident. If any tampering occurs to the chain or an individual proof, it should be detectable.
- Validation: A provenance chain can be used to validate the location visit claims along with their chronological order.
- Non-repudiation: The provenance chain must be able to reveal the true travel path of the prover in case of denial or re-ordering of location visits.

### 1.3. Challenges of Location Proofs System

To design a secure location proof system, the following are the challenges [5,9]:

- Integrity: The prover should not be able to create a fake location proof alone or by collaboration with other entities. The prover may adopt relaying.
- Collusion Resistance: In the distributed model, location authorities aid in the proof generation, and witnesses endorse them. However, both location authorities and witnesses are not guaranteed to be trustable. The user can collude with location authorities and witnesses to generate false location proofs. A location proof system must be capable of collusion resistance.
- Storage: It must be determined as to where proofs should be stored. Storing all proofs in a central server will result in bottlenecks. In the distributed modal, each location may have common storage for location proofs; nevertheless, the verification process becomes difficult to access the proofs from distributed locations. Storing the location proofs over the user’s smart device is best if the user is trustable. However, the user has full control over his device and can manipulate the proofs; thus, proofs must be tamper-evident.
- Clock synchronization and timestamping: LPS is supposed to maintain the sequence of locations visited by the prover to allow the auditor to verify the travel path. To track the chronological order of visits, clocks of entities in the distributed environment must be synchronized. That is, the clocks of the LA, any witness and the prover need to be synchronized. Timestamps reported by the prover cannot necessarily be trusted; therefore, a reliable source providing the true timestamps for location proofs is needed.
- Location Privacy: The location privacy concern of the prover can be seen from two perspectives. First, the prover wants the auditor to validate the location proofs of the prover but wants to limit the amount of information exposed to the auditor. Secondly, the prover, while generating a location proof, needs attestation from a witness co-located. Hence, the prover wants the witness to endorse the location proofs without revealing their real identity. So, location privacy determines the level of granularity of location information; the user wants to share with auditors on one hand and wants the witnesses to endorse the location proof anonymously.
- Deployment: From a deployment perspective, a location proof system should be incremental. In the beginning, a limited number of location authorities could be deployed, but later on, adding new location authorities to divide a large area into small zones must be easy.
- Unforgeable Identities: Entities involved in the location proof system must have unique and unforgeable identities. Otherwise, a prover may generate false identities to masquerade as dummy witnesses to obtain a false location proof. The identity of an entity in the system must be linkable to a single party while keeping its anonymity for other entities in the system to preserve its privacy.
- Proof Generation Time [13]: The proof generation time is the interval between the request generated by the user and the final proof generated by the system. It should be short enough (a few seconds) for the system to be practically usable. There is a trade-off between security and proof generation time. However, higher security guarantees requiring a longer proof generation time may make the system inapplicable in real-world scenarios.
- Proof Size [5]: Location proof size becomes a constraint for location proof systems, bearing in mind the storage capacity of smart devices and location authority especially when location provenance is supported. Additionally, space requirements change when location privacy is enabled and support granularity levels for location information with respect to entities in the system.
- Number of Entities/Witnesses Involved [9,13]: Distributed location proof systems in general, and any witness-oriented location protocol in particlazzur, involve two or more entities in the proof protocol. The number of entities involved in location proof protocol provides the reliability of the physical presence of a user at a location. However, having more entities in the protocol provides reliability at the cost of proof generation time.

The trustworthiness of a location proof demands a cheat-proof system [30]. Since the user has full control over his smart device, they are able to lie about the position or can tamper with the proof after its generation. Another possibility is that the participants may collude to generate fake proof of physical presence. In the existing literature, outlier detection techniques, an entropy-based trust model, and witness-asserted location proofs have been used to mitigate collusion attacks to some extent. However, to the best of our knowledge, three-way collusion has not yet been addressed by any existing witness-oriented schemes. Three-way collusion occurs when a prover, witness, and location authority (LA) are all malicious and collude to generate a false proof of location. In the literature two types of weaknesses have been identified such that if any of these exists, then three-way collusion is possible:

1. Participant selection decision control lies with participants of the protocol: A participant’s selection control for proof generation lies with the user who will always choose the colluding participant to cheat. Xinlei et al. [9] proposed the STAMP scheme, which relies on witness selection on the peer discovery mechanism provided by the underlying communication technology of the user’s device. Similarly, Brambilla et al. [26] developed a scheme that allows the user to select the peer for assistance in a proof generation. In WORAL [13], a user looks up the location authority, which is the stationary entity on a site, which randomly chooses a witness from a “witness list” for aiding the user in a proof generation.
2. Participant selection decision control lies with the single party: The user has direct communication with LA, and both can collude to generate a false proof. Even in the witness-oriented scheme, witness selection control either lies with the prover or LA. So, if both the prover and LA are colluding, it will be easy for them to choose a colluding witness. In the scheme of Rasib et al. [3], LA is the stationary entity on a site, which randomly chooses a witness from the “witness list” for aiding the user in a proof generation. Furthermore, the puppet witness attack and wormhole attacks are easy in this scenario.

Our proposed MobChain scheme is designed to mitigate these weaknesses and to provide better resistance against the three-way collusion problem of existing schemes.

Our Contribution: The key contributions of this paper are as follows:

1. To the best of our knowledge, MobChain is the first three-way collusion-resistant witness-oriented LPS. We highlighted the two weaknesses in the design of existing schemes enabling three-way collusion attacks. Furthermore, we proposed the distributed consensus strategy in MobChain to mitigate the design weakness of existing schemes and resist three-way collusion.
2. A schematic description of distributed consensus and location proof generation protocol providing coarse-grained messages and communication to achieve distributed consensus and location proofs.
3. We provide a detailed security analysis of MobChain highlighting the possibilities of fake proof generation and permutations of collusion attacks and how the proposed scheme provides resistance.
4. Experimental evaluation determines the low bounds on space and computation requirements of MobChain. Furthermore, it highlights the impact of the distribution of entities in the system. That is,
  - Impact of the number of active workers (witnesses and location authorities) on
    - Decentralized Decision Time;
    - Proof Generation Time (PGT); and
    - Decision Block Size and Location Proof Size.
  - Impact of the number of entities participating in distributed consensus on decision block size and proof generation time.
  - Concurrent request impact on decentralized decisions time.

Organization of Paper: The rest of the paper is organized as follows: In Section 2, a detailed overview of the related work in this domain is provided. In Section 3, we highlight the threat model and the assumptions about the adversary. In Section 4, we provide an overview of the proposed MobChain scheme and architecture. In Section 5, we provide the detailed security analysis of the proposed scheme. The experimental evaluation of the proposed architecture is provided in Section 6. In Section 7, we conclude the paper and highlight areas for improvement as future work.

## 2. Related Work

In this section, we review the existing work on LPS. Generally speaking, in location proof systems, a prover is the main entity that wants to generate secure location proof for his or her physical presence at a particular time instance through secure localization [2,9]. Localization is a mechanism by which a smart device can identify its position relative to some other smart device, map, or global coordinate system [6]. Location-aware schemes rely on either software-based or hardware-based localization techniques. Gabber et al. [31] have incorporated multiple channels (GPS, cellular telephony, Caller-ID, satellite, etc.) to monitor the movement and location of smart devices. However, it has been proved later that a malicious entity can bypass such a multi-channel combination approach [32]. For example, GPS signatures [33] were prone to spoofing attacks [34]. Bauer et al. [35] have discussed the vulnerabilities of wireless-based localization approaches against non-cryptographic attacks using a low-cost antenna. Gruteser et al. [36] have proposed an anonymity-based privacy-preserving localization technique. This scheme is based on middleware to adjust the location information along spatial–temporal dimensions for a centralized location broker service. Zugenmaier et al. [37] have introduced the “location stamps” concept utilizing cell phones. Dominik et al. [38] have proposed a secure and tamper-resistance location proof system based on visual features and image recognition without overburdening the user.

Researchers have also explored hardware-oriented localization schemes. Hardware-based localization techniques include measuring signal attenuation [34], measurement of round-trip time (RTT) [11], and voice signatures [39]. However, these approaches have failed to provide secure localization under adversarial settings. In [34], the secure positioning of wireless devices under adversarial settings has been discussed. Analysis of positioning algorithms (including received signal strength (RSS), ultrasound time-of-flight (TOF), radio TOF, civilian GPS) against position and distance spoofing attacks is performed providing the vulnerabilities details. Signal attenuation techniques suffer from channel noise and the constraint of line-of-sight makes them difficult in practical scenarios [13]. Saroiu et al. [40] have used a trusted hardware trusted platform module (TPM) and virtual machine-based attestation to make the sensor readings trustable. Similarly, Gilbert et al. [41] have proposed a TPM-based trustworthy mobile sensing platform to provide data integrity and privacy protection.

Furthermore, Luo et al. [32] have proposed six design goals for a proactive location proof system providing privacy protection. Saroiu et al. [42] have devised a Wi-Fi-based protocol, where Wi-Fi access points (AP) aid the prover in the generation of trusted location proofs. However, their scheme is prone to collusion attacks, as an AP and prover can collude to generate fake location proofs. User privacy has been the primary concern in [42], as the real identity of the user was exposed to AP. The authors in [42] have described the security properties of secure location proofs and have discussed the applications where LBS with incentives provide a motive for users to lie about the location. The VeriPlace [43] is a privacy-preserving LPS with collusion resistance support. However, the assumption of the short interval between location proofs for collusion detection makes the VeriPlace vulnerable. If the interval between two chronologically close location proofs is not close enough, then the VeriPlace will treat them as suspicious. Therefore, VeriPlace puts the burden on the user to have frequent location proofs. All these schemes have ignored the chronological order of location proofs. Hassan et al. [5] have designed a scheme for location proofs with wireless access points as location authorities and co-located smart devices designated as witnesses for proofs endorsement through Bluetooth. This scheme has removed the dependency on the trusted third parties. Nonetheless, location provenance was maintained by [5] to record the chronological order of location proofs, which was missing in previous schemes. All the above schemes are under the category of centralized architectures. 

In the distributed model, Davis et al. [44] have devised a scheme to generate location proofs with the help of smart devices within proximity. However, the scheme [44] was not collusion resistant. Zhu et al. have proposed an LPS called “APPLAUS” [7] (i.e., A Privacy-Preserving LocAtion proof Updating System), which has utilized the Bluetooth technology allowing the co-located devices to generate the location proofs mutually. *APPLAUS* has utilized “pseudonyms” for privacy preservation. However, communication overhead is introduced due to the generation of dummy proofs periodically. Moreover, to improve the security of APPLAUS, the authors have devised a collusion detection mechanism based on the ranking and correlation clustering approaches. However, the adopted collusion detection mechanism was later proved to be energy inefficient, and a successful detection ratio >0.9 was possible only when the collusion percentage is <0.1. Ananthanarayanan et al. [45] have introduced a framework called “StarTrack” enabling tracks of information holding data about a person’s location, time, and metadata. However, this concept was quite similar to the location provenance chain, as data were recorded in the time-ordered sequence. No security measures were taken, thus leaving the scheme vulnerable to malicious user manipulations. Gonzalez-Tablas et al. [46] have presented the notion of “*Path-stamps*”, extending the concept of “*location-stamps*” [37] by recording the history of the visited location’s proofs in a hash chain. Rasib et al. [3] have relied on a WiFi-enabled smart device to generate the location proof and have also proposed the formal requirements for the design of LPS. The scheme [3] treated the location authority to be malicious and empowered the LPS with witness endorsement. Additionally, the authors have highlighted the possible attacks and devised a trust score mechanism to evaluate the reliability of witnesses providing the collision resistance. However, none of these schemes described the requirements for a secure location provenance mechanism formally. In *OTIT* [29], for the first time, the requirements of secure location provenance have been formally defined. Furthermore, the authors have also performed a comparative analysis of different techniques used to maintain a provenance chain. The comparison has been based on provenance generation time, sequential verification time, sparse verification time, and space requirement. Wang et al. [9] have proposed “*STAMP*”, which is a spatial–temporal provenance assurance with the mutual proofs scheme. STAMP ensures a user’s privacy while providing the integrity and non-transferability of location proofs. To guard against collusion attacks, an entropy-based trust model is utilized. Furthermore, STAMP reduced the dependency on multiple trusted parties to a single semi-trusted party i.e., certification authority (CA). The scheme has also supported the granularity level control for the exposer of location information to the verifier by the user. STAMP is the first scheme to deal with two collusion attacks. (i) User-A is physically present at a target location and generates a false proof for User-B by masquerading. (ii) Terrorist Fraud Attack where two malicious users collude to generate a fake proof of location for each other. To prevent terrorist fraud attacks, the Bussard–Bagga distance bounding protocol [47] has been used with a trade-off on performance in STAMP. Later on, Hasan et al. [13] proposed “Witness ORiented Asserted Location provenance (WORAL)”, which is a distributed witness-oriented secure location provenance framework for mobile devices. WORAL is a complete working system built by integrating the *asserted location proof (ALP)* (proposed in the scheme [3]) and OTIT [29] model for managing secure provenance. WORAL has provided collusion models and corresponding threats. Furthermore, the authors have also claimed that the system is only 12.5% vulnerable because of the inability to resist three-way collusion. WORAL has evaluated the protocol based on characteristics including the proof generation time, maximum distance threshold (depending on localization technique), proof size, number of participants of protocol, collusion detection rate. WORAL has established the vulnerability matrix to ensure that fake proof generation is not possible in any scenario. For privacy preservation, *crypto-ids* has been used by WORAL, such that the many-to-one relationship holds between crypto-ids and the real identity of the user. 

Moreover, Wenbo et al. [48] have discussed the cheating possibilities of users against the location verification mechanism used by FourSquare. Authors crawled the website and performed analysis on the crawled data to highlight the vulnerabilities that are exploitable for cheating on the user’s location. Another dimension of research based on location information is quantum-based geo-encryption [49], which enhances the security of a traditional cryptosystem by introducing a “geolock” in which the encrypted data can only be decrypted at a specific location. The motive of quantum-based geo-encryption is to reduce the chances of spoofing attacks to zero as the adversary gets away from the targeted location. Brassil et al. [50] have proposed a robust location authentication mechanism while relying on the femtocells of 802.11x access points. The basic idea was to analyze the traffic signatures for location verification to make the scheme device-independent and carrier-independent (3G, LTE, etc.), which was efficient due to the use of the non-cryptographic technique for location verification. Idrees et al. [51,52] have proposed secure provenance schemes in a distributed environment using aggregated signatures. Furthermore, the authors did not assume any transitive trust among consecutive colluding users, which is a stronger security model compared to previous studies. However, the proposed scheme is not intended for providing secure location proofs. A detailed review of trustworthy data using provenance in various domains is provided by the authors in [53]. The authors have explored the notion of trust using secure provenance in various domains such as wireless sensor networks, cloud computing, and databases. A comparative analysis of essential security properties is also provided for various secure provenance schemes in these domains. QuietPlace [51], an ultrasound-based location proof system, is introduced and provides strong identities. Kounas et al. [51] claim to provide strong user privacy using an ultrasound-based approach while proving the physical presence of a user at a claimed location.

Brambilla et al. [26] have proposed the first decentralized location proof system by using blockchain for P2P overlay schemes. However, participant selection control is in the hands of the prover; therefore, decentralization standalone still makes their scheme vulnerable to collusion attack. The proof generation protocol of the scheme [26] allows the direct communication between the prover and responding peer; therefore, the possibility exists that both can collude. However, the scheme can resist backdating and future dating attacks. Amoretti et al. [27] have proposed a blockchain-based LPS considering both static and dynamic entities. Nasrulin et al. [28] have also proposed the decentralized location proof system and evaluated its performance and security by developing the POC for supply-chain management. Similarly, Nosouhi et al. [52] proposed a privacy-aware, blockchain-based secure location proof system that claims to resist the P-P collusion and P-W collusion attacks. However, the scheme in [52] is vulnerable to multiple-party collusion attacks. Therefore, all existing centralized, distributed, and decentralized location proof systems are unable to resist three-way collusion attacks. PASPORT [53] was a blockchain-based decentralized location proof system with a claim to resist P-P collusion and P-W collusion attacks while removing the dependency on any trusted third party. PASPORT takes the witness selection control from the prover; however, it is still unable to resist a three-way/multi-party collusion attack as the verifier assigns the witness to the prover for location proof generation.

## 3. Threat Model

In our threat model, we discussed the adversary’s roles and capabilities. We also discuss the possible attacks by individuals and colluding malicious parties of the system. The primary assets of the MobChain that are vulnerable to attacks by malicious participants include the following:

- Decision Block: Block generated by a consensus of supervisor nodes for the selection of location authority and witnesses to aid the prover for proof generation.
- Decision Blockchain: Blockchain records the decision blocks generated by the consensus of supervisor nodes in chronological order.
- Location Proof: A proof of the presence of the user at a location with an exact timestamp.
- Location Provenance Blockchain: The blockchain records all the past location proofs in a chain to keep their chronological order intact.

To the best of our knowledge, currently, no location proof system has considered three-way collusion, i.e., all three participants (Prover, Witness, Location Authority) involved in a proof generation can be malicious and colluding at the same instance. For the security analysis of MobChain, we have considered the following possible attacks discussed in the literature [3,9,13]:

1. False Presence: A malicious prover may want to get fake proof of location without being physically present at the claimed location.
2. False Time (back-dating, future-dating): The prover tries to get the proof for his true location with a timestamp different from the time of visit. Participants of the protocol collude to generate the location proof for the prover with a past timestamp in the back-dating attack and with a future timestamp in the future-dating attack.
3. Sequence Alteration: In a sequence alteration attack, the prover tries to present a false travel path by changing the chronological order of location proofs.
4. Implication: Participants of the protocol dishonestly prove the physical presence of the prover at the location to victimize the prover.
5. False Endorsement: In the witness-oriented model, the witness colludes with the prover to falsely endorse that prover is physically present at the claimed location.
6. Presence Repudiation: The user tries to deny his presence at a location at a time instance for which the location proof has been generated.
7. Proof Tampering: A legitimate old proof’s timestamp or location information may be modified to present it as a new proof.
8. Puppet Witness Attack: The location authority and prover may collude and create a puppet witness to falsely endorse the proof of location or relay the request to a remote witness not co-located to the prover at the time of visit.
9. Wormhole Attack: Any entity of the system physically present on the desired location may impersonate the prover and generate the location proof for him. It is not necessary that the prover has shared his secret keys with the impersonating party but might have established a covert communication channel, and the impersonator might be relaying the messages of proof generation protocol to the prover. A wormhole attack is also known as a terrorist fraud attack.

### 3.1. Assumptions: We Make the Following Assumptions about the Actors in the System

1. Participants of the system are well known to the system, and they do not share their private keys.
2. Users do not have multiple identities to launch a Sybil attack.
3. Smart devices are not shared with other participants.
4. At least one witness is available at the location of the visit.
5. No entity in the system will be able to compromise 51% of the supervisor nodes [27].

### 3.2. Goals

#### MobChain has the Following Goals

- To provide resistance to three-way collusion while ensuring protection against known attacks over existing schemes.
- To measure the impact of introducing distributed consensus in location proof generation protocol.
- To analyze the storage requirements for peers (supervisor nodes) for maintaining the blockchain.

## 4. MobChain 

Overview and motivation: Building a secure location proof system capable of collusion resistance is a real challenge, since the participants of the protocol are not necessarily trustable and are in full control of their smart devices. In the given circumstances, if participant selection control lies with either the prover or the designated authority, then collusion is possible. MobChain extends the design of WORAL [13] by introducing decentralized decision making for the selection of participants (LA, Witness) for the location proof generation. WORAL treats the location authority as malicious as are the witness and prover. 

Since in practical situations, all three participants (prover, location authority, and witness) can be malicious and colluding a location proof system cannot guarantee the reliability of location proofs assuming the location authority as trusted. Therefore, WORAL is assumed to provide better security in the context of location proofs. However, the weakness of the WORAL scheme is the strong assumption that all participants will not collude at the same time in a location proof protocol. The inability of WORAL to resist three-way collusion serves as a base for our problem statement. 

To improve the security of location proof systems, our contribution is the proposal of two design principles to mitigate these weaknesses:

- Separation of participant selection control from location proof generation protocol
- Decentralization of control decisions (selection of participants, the addition of location proofs in provenance chain, validation of location proofs on the request of the third party).

By separating the participant selection control from the location proof generation protocol makes collusion hard for malicious participants, since a third party will decide who will be assisting the prover for proof generation. However, delegating the participant selection control to a single third party still presents collusion problems if thay are assumed malicious. Therefore, we adopted the decentralized control-decision strategy in our proposed scheme to make the collusion harder for participants. Decentralized decisions will demand the malicious participants to compromise the majority of the decision makers to manipulate the decision in their favor to generate a fake proof [27]. 

In MobChain, the provers and witnesses are mobile entities visiting a location temporarily, while the location authority is a static entity designated on the location permanently. Therefore, the location of the location authority is known, while mobile entities visiting the location report their location to the MobChain P2P network (i.e., the admin layer established by supervisor nodes). Any mobile entity visiting the location can take on the role of prover or witness. Once a mobile entity requests proof of location, it becomes the prover for that request, while all other entities co-located on the location are eligible to become a witness for that prover. For some other instance of time, this witness can become the prover on requesting the proof of its location, and the prover can become the witness. 

Furthermore, all the supervisor nodes in the admin layer are eligible to receive location proof requests from visiting mobile entities. The admin layer node on receiving the location request from the visiting entity broadcasts the message in the admin layer to let other nodes know about the presence of a mobile entity. Mobile entities keep the admin layer updated about their presence and exact location through a periodic ping mechanism to remain eligible for the selection as a witness. The witness selection criteria include two parameters for mobile entities (i) uptime and (ii) number of requests entertained. ‘*Uptime*’ for mobile entities is calculated using the time difference of the first location request and last ping time. If the mobile entity’s ping request is not received within a certain time, then it is removed from the list of available mobile entities. Later, when the mobile entity comes back to the location, then it is considered as a new entity. 

Another promising aspect of MobChain is utilizing blockchain capabilities to support location provenance. Location provenance provides the history of locations visited by maintaining the tamper-evident chain of all location proofs generated in the past while keeping their chronological order intact. Blockchain is a public distributed ledger, which stores the transaction data in a modification-resistant chain [15]. Since blockchain is implicitly tamper-resistant and maintains the chronological order of data inserted, therefore, it is an ideal candidate to maintain location provenance. Additionally, blockchain removes the single point of failure, as all peers of the blockchain hold the data and can validate it on request. Peers in the P2P network of the blockchain establish distributed consensus before committing any data over the blockchain. In non-blockchain-based location proof systems, location provenance is either maintained by the location authority or saved on the prover’s smart device. If location provenance is maintained on the user’s smart device only, then in case of damage or theft, all location proofs will be lost. If location provenance is maintained by the location authority, even then, it remains the single point of failure and can become a bottleneck in case of high loads for location proofs verification. To overcome these limitations, blockchain is ideal for maintaining location provenance. However, the primary difference between blockchain and MobChain is that in traditional blockchains, the decentralization focus is on establishing a distributed consensus about whether to make any new data block part of the blockchain or not. While in MobChain, the decentralization primary focus is on establishing distributed consensus for control decisions, including the following:

- Approval of witness and location authority for the prover to start location proof generation.
- Making an approval decision block part of the decision blockchain (maintained by the admin layer of MobChain).
- Making a generated location proof part of a location provenance chain (maintained by the admin layer of MobChain).
- Validation of old location proofs on the request of the third party involving a decision blockchain and location provenance chain.

For location-privacy preservation, we adopted the crypto-id based pseudonyms approach of WORAL for MobChain. Supervisor nodes can only validate the prover based on its crypto-id to ensure that it is a valid user of the system. However, supervisor nodes cannot link their location information to the personal identity behind that crypto-id to violate the privacy of the user.

### 4.1. Architecture Overview 

Before explaining the MobChain architecture, we need to elaborate on certain abbreviations and terminologies. A *worker node (WN)* is a general category that encompasses both the location authority and witnesses. The *supervisor node (SN)* refers to the individual peer that is part of a P2P network of the MobChain admin layer. The *request-receiving supervisor node (RRSN)* is the supervisor node that receives the *proof request (PReq)* from the prover and initiates the distributed consensus for the location proof protocol participant’s selection. However, all the supervisor nodes in the admin layer can become RRSN for different provers. *RRSN* is not a designated role for any specific supervisor node. We labeled the request-receiving supervisor node *RRSN* to differentiate it from other supervisor nodes in the schematic description of MobChain working in Section 4.2. *Decision block (DB)* is the final decision message created by *RRSN* when the distributed consensus protocol ends. The *RRSN* then sends the approval message, which includes the decision block reference such that it allows the prover, location authority, and witness to initiate the location proof generation. Table 1 summarizes the abbreviations.

To incorporate the two aforementioned design principles, MobChain is virtually organized into two layers.

1. Admin Layer: All control decisions are the responsibility of the admin layer underlying the P2P network. Commodity devices in the admin layer form a P2P network of supervisor nodes. Supervisor nodes take proof requests from the prover, and all control decisions are taken through a distributed consensus mechanism within the admin layer. The primary operations of the admin layer are as follows:
  - Distributed consensus (decentralized decision for the selection of worker nodes co-located to the prover for assistance in location proof generation).
  - Final validation of location proof generated and adding it to blockchain to build location provenance.
  - Auditor role to verify the location proof requested by the third party.
2. Service Layer: In MobChain, the service layer is not part of the P2P network admin layer. The service layer consists of all devices (witness, prover, and location authority devices) that may participate in the location proof protocol. The location authority is the static entity permanently designated at the location while witnesses and provers are mobile entities who may visit the location temporarily. Any mobile entity visiting the location can be in the role of a prover and witness. Once a mobile entity requests the proof of location, it becomes the prover for that request, while all other entities co-located on the location are eligible to become a witness for that prover. At some other instance of time, this witness can become the prover on requesting the proof of its location, and the prover can become the witness for him in the location proof protocol. Since the location authority is the static entity designated by the system, therefore, its location is known in advance. Furthermore, it is responsible for ensuring that the prover and witnesses are physically present at the location. All mobile entities visiting the location connect to supervisor nodes in the admin layer to let the system know their presence at the location. No permanent connection is required with the admin layer by these entities. However, a periodic ping mechanism ensures the admin layer is informed about their presence and location. Once any of these mobile entities request the system to provide location proof, it becomes the prover, and other co-located entities are considered available witnesses. RRSN pings the witness selected after distributed consensus to ensure that it is available and then sends the approval message to the prover to initiate the location proof protocol with the chosen witness and location authority.

Two types of blockchains are maintained by the admin layer of MobChain:

1. Decisions Blockchain: Tracks all decisions taken by the admin layer, keeping their chronological order intact with timestamps. Blocks of the decision chain will serve for validation of location proofs and cross-checking of the witness elected by the admin layer and the actual witness who aided in proof generation. It will also provide protection against future and backdating attacks and three-way collusion for a fake proof generation or representation of valid old proof after tampering.
2. Location Provenance Blockchain: All location proofs of the prover will be recorded in chronological order in the location provenance chain.

**Figure 1:**
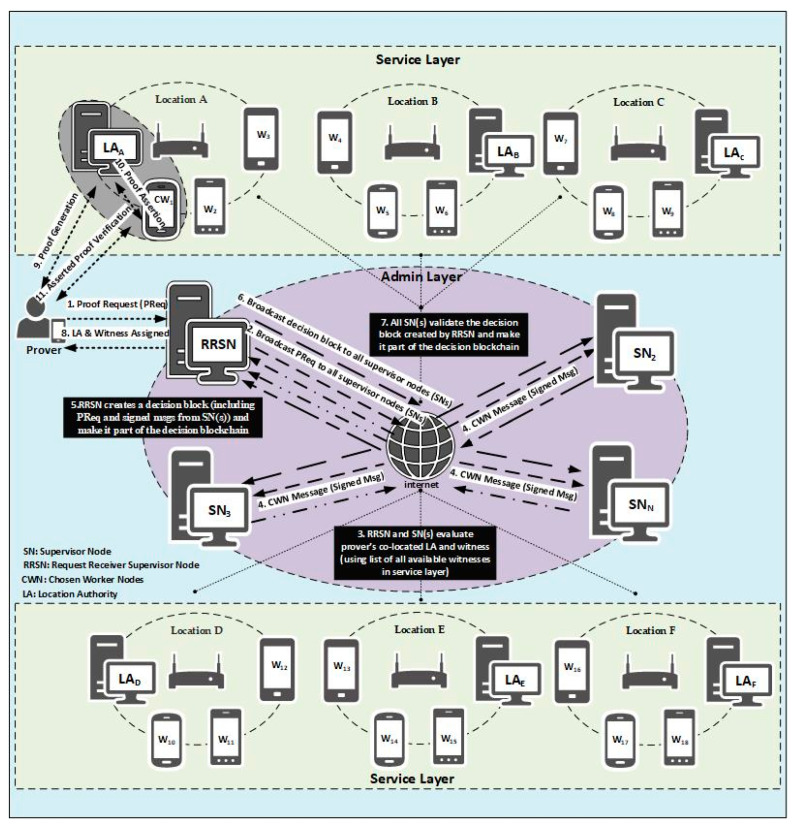
MobChain architecture.

As shown in Figure 1, all supervisor nodes will be deployed at multiple geographical locations, and the initial P2P network will be established. Later, public addresses of supervisor nodes will be published, so that worker nodes can join the network. Worker nodes will authenticate with the supervisor node to join the P2P network of MobChain. The geolocation of the newly joining worker node will be broadcasted among supervisor nodes. Thus, a common list of available worker nodes with their geolocations is established in the admin layer of MobChain. Geolocations of worker nodes can be refreshed on demand and at regular intervals. Supervisor nodes will use geolocations from the worker nodes list to find the prover’s co-located worker nodes to establish a distributed consensus.

### 4.2. Distributed Consensus 

Decentralizing the decisions demands a mechanism to establish a consensus on the same value by the underlying P2P network. In MobChain, the decentralized decision of selecting worker nodes (*LA* and Witness) for the prover is achieved through a specialized consensus mechanism, as described in Figure 1. The consensus mechanism is started by *RRSN* on receiving the proof generation request. *RRSN* generates the specialized message including the proof request from the prover and broadcasts it in the admin layer. On receiving the specialized request message from *RRSN*, all supervisor nodes evaluate the prover’s co-located workers (witnesses and location authorities) using the geo-coordinates of the prover and available worker nodes. Once co-located workers (LA and witness) are evaluated, a signed decision message (*CWN*) is sent to *RRSN* by every supervisor node. *RRSN* will wait until the consensus threshold is satisfied; i.e., (*N*/2) + K signed decision messages agreeing on the same witness and location authority are received. *N* is the number of supervisor nodes and *K* is the percentage of decision messages above 50% agreeing on the same witness and location authority. The consensus threshold value is a trade-off between the reliability of the decision and the decision time. The higher the consensus threshold, the more reliable the decision will be but the higher the decision time will be as well. Therefore, we have chosen the (*N*/2) + 1 consensus threshold value to balance this trade-off. Once the consensus threshold is accomplished, the *RRSN* will generate a decision block by including all the signed messages received. Then, the finalized decision block will be broadcasted in the admin layer by *RRSN*. The decision block generated is passed to the prover and will be used in a generated location proof. All supervisor nodes will validate their signed message and the original request from the prover in the decision block to make it part of the decision blockchain. If validation of the decision block fails, then the supervisor nodes will discard it. Later on, the location proof referring to the rejected decision block will be treated as fake and discarded. Only those location proofs will be made part of the location provenance chain for which a valid decision block will be present in the decision blockchain. 

### 4.3. Schematic Description of Distributed Consensus and Location Proof Generation in MobChain

The working of MobChain is divided into two phases.

- Decentralized Decision on Witness and LA Selection (Distributed Consensus) Phase: On a prover’s request, the *RRSN* initiates the protocol over the admin layer such that the witness and *LA* are elected through a distributed consensus of supervisor nodes.
- Witness-Oriented Location Proof Generation Phase: Once the *LA* and witness are intimated to assist the prover, the actual location proof generation phase starts.

To explain the working of the location proof generation protocol, we are using the schematic description of the messages communicated in each step of both phases. 

#### 4.3.1. Decentralized Decision on Witness and LA Selection (Distributed Consensus)

The protocol initiates with *PReq* sent by the prover to any supervisor node, which is represented as *RRSN*:

*Req* = *[ID_P_*, *T_P_*, *L_P_]* (1a)

*PReq* = *[Req*, *Sign_P_(Req)]*. (1b)

In Equation (1a), *ID_P_* is the unique identifier of the prover, *T_P_* is the timestamp of the prover’s smart device, *L_P_* is the current location of the prover, and *Sign_P_(Req)* will protect against the repudiation of the prover. To present that the prover at location *L_P_* has requested for proof generation, *RRSN* constructs a message *PReq′* and broadcasts it to all other *SN*(s):

*PReq*′ = *[PReq, T_RRSN_, Sign_RRSN_(T_RRSN_), ID_RRSN_]*. (2)

In Equation (2), *ID_RRSN_* is the unique identifier of *RRSN*, and *T_RRSN_* is the timestamp of the *RRSN* machine when it received the *PReq*. T_RRSN_ indicates the freshness of the message, and *Sign (T_RRSN_)* helps the other *SN*(s) to ensure that the request is from the known valid supervisor node of the system. Thus, *PReq′* will help mitigate replay attacks, as any message with *T_RRSN_* older than a certain threshold duration will be discarded. On receiving the message from *RRSN*, every *SN* validates the *PReq′* and then finds the prover’s co-located witness and *LA*. For this purpose, *SN*(s) computes the priority for all available witnesses and *LA*(s) registered in the network using the below expression,

(A) $$Priority=\frac{R_{P}\times T_{U}}{D_{\left(P-W\right)}}$$

In Equation (A), *R_P_* is the number of proof requests entertained by the worker node, i.e., witness or LA, *T_U_* is uptime, and *D_(P−W)_* is the distance (<short-range communication technology) between the prover and worker node. The number of requests entertained is assumed to be greater than 0 and will help in rotating the chance of participation by all entities in proof generation, whereas uptime is the measure of the reliability of the witness and location authority. To elaborate on the impact of priority calculation expression on the security of MobChain, suppose the *RRSN* is compromised and colludes with the prover to make the centralized decision in favor of a malicious witness who is willing to collude with the prover. If we see the priority calculation equation, the number of requests entertained (by the witness and location authority) and uptime are two parameters that enable honest supervisor nodes to prevent the MobChain against the compromised supervisor node, since the number of requests entertained by the witness and the location authority is the count of old location proofs in which the witnesses and location authority individually participated. Therefore, several requests for entertainment information cannot be modified by the compromised supervisor node in the admin layer. The second parameter is the uptime of the witness and the location authority, which is the difference between the first request to the admin layer (to notify its presence and location) and the last ping time. Therefore, the uptime value of any witness and the location authority cannot be manipulated by any single supervisor node in the admin layer. Another possibility for a compromised supervisor node is to generate the approval decision of prover choice, contradicting the choice of witness and the location authority of other supervisor nodes. In this scenario, when a decision block will be propagated in the admin layer, then honest supervisor nodes will validate it and reject it, and later, the location proof generated against this decision block will also be rejected. Hence, MobChain remains secure until 51% of the supervisor nodes are compromised by the prover.

The large value of uptime indicates that the witness and the location authority are more trustable and reliable. Uptime will help in mitigating the puppet witness attacks and *Sybil* attacks. We can also specify the minimum threshold for the uptime of worker nodes to become eligible for participation in proof generation, making the puppet witness attack and *Sybil* attacks harder. Once the priority is calculated for all, the witnesses and *LA* with higher priority are chosen, and every *SN* informs the *RRSN* about his choice by sending a decision acknowledgement message ‘*DAM*’ i.e.,

*DM_SNi_ = [ID_SNi_, T_SNi_, W_C_, LA_C_, PReq′]* (3a)

*DAM_SNi_ = [DM_SNi_, Sign_SNi_(DM_SNi_)]*. (3b)

In Equation (3a), *ID_SNi_* is the unique identifier of *SN_i_*, *T_SNi_* is the timestamp of the *SN_i_* machine, *W_C_* is the witness chosen, and *LA_C_* is the location authority chosen by *SN_i_* for assisting the prover in a proof generation. While *Sign_SN_(DM_SN_)* serves the non-repudiation of *SN*(s) and later will be used to validate the final decision block constructed by *RRSN*:

*DB_i_ = [{DAM_SNi_, …, DAM_SNN_}, W_C_, LA_C_, ID_RRSN_, T_DBi_]* (4a)

*DB′_i_ = [DB_i_, Sign_RRSN_(DB_i_)]*. (4b)

In Equation (4a), the final decision block *‘DB′’* incorporates all *DAM* messages received from *SN(s)*, the *W_C_* and *LA_C_* are the chosen witness and location authority, respectively, over which more than 50% of *SN(s)* have consensus, *ID_RRSN_* identifies the creator of the block, and *T_DBi_* is the time of the creation of the block. To make the new decision block part of the decision block chain, its unique ID *‘ID_DBi_’* is calculated:

*ID_DB′i_ = H(H(DB′_i−1_), DB′_i_)*. (5)

Now, the *RRSN* will broadcast the new decision block to all *SN*(s); furthermore, it will construct an approval message *‘AMsg’* for prover containing the decision block ID, chosen witness, and location authority:

*AMsg = [PReq, ID_DB′i_, W_C_, LA_C_, T_AMsg_]* (6a)

*AMsg′ = [AMsg, Sign_RRSN_(AMsg)]*. (6b)

In Equation (6a), *T_AMsg_* is the time of the creation of the approval message, and in Equation (6b), the approval message constructed in (6a) is signed by *RRSN* so that the prover can validate it to ensure that the message is from a valid *SN*.

#### 4.3.2. Witness-Oriented Location Proof Generation Phase

**Figure 2:**
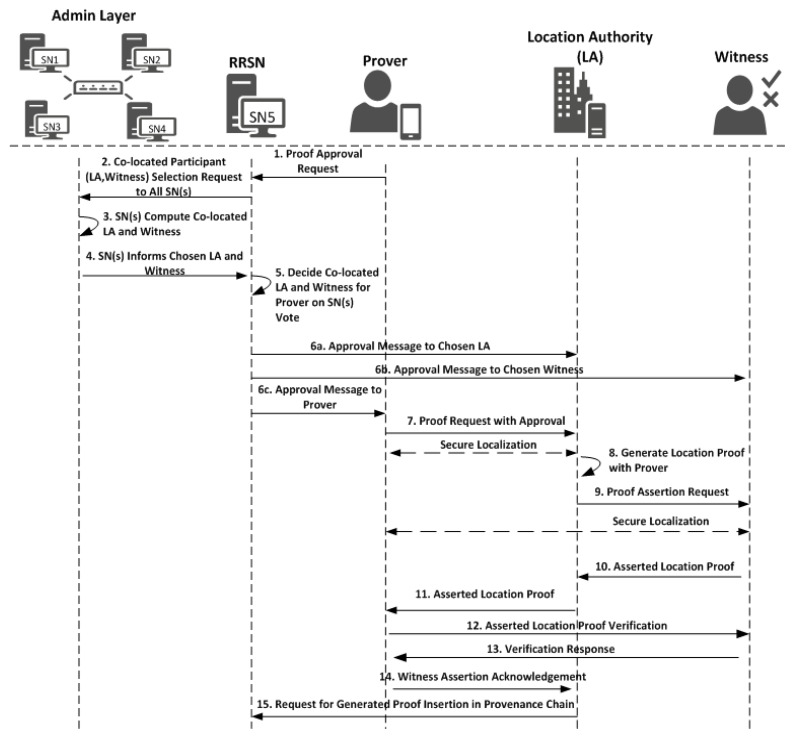
Witness-oriented location proof protocol.

Working of the witness-oriented location proof generation protocol described in Figure 2 is as follows:

1. The prover requests the location authority *LA_C_* (chosen by the admin layer) to assist him in proof generation by sending an *LPReq* message.
  *LPReq = [AMsg′, T′P]* (7)
  where *AMsg′* in Equation (6b) is the approval message from the admin layer after distributed consensus, which is sent by *RRSN*, and *T′_P_* is the time of the request to *LA_C_* by the prover.
2. *LA_C_* first validates the *LPReq* against the *AMsg′* received from *RRSN*. Once validated, it then performs secure localization to ensure that the prover is physically present at the mentioned location. Finally, *LA_C_* generates the location proof *LP* against *LPReq*,
  *LP = [ID_LA_, LPReq, T_LS_]* (8)
  where *T_LS_* is the time of location statement generation.
3. *LA_C_* then creates the location proof assertion request “*AReq_LP_*” and sends it to witness *W_C_*.
  *AReq_LP_ = [LP, Sign_LA_(LP)]* (9)
4. The witness validates the *AReq_LP_* against *AMsg′* to ensure that *LA* is asking for the assertion of validity and then performs the secure localization to ensure that the location authority and prover are not colluding and the prover is physically present on the reported location. Once verification is successful, the witness creates the assertion statement “*AStat*”
  *AStat = [ID_DB′i_, ID_P_, ID_LAC_, ID_WC_, H(AReq_LP_), T_AStat_]* (10)
  where *ID_DB′i_* is the decision block ID, *ID_P_* is the ID of the prover, *ID_LAc_* is the ID of the chosen location authority, *ID_Wc_* is the ID of the chosen witness, *H(AReq_LP_)* is the hash of assertion request message, and *T_AStat_* is the time of assertion statement generation. Finally, the witness generates the assertion response “*AR*” and sends it back to the location authority.
  *AR = [AStat, Sign_Wc_ (AStat)]* (11)
  The witness now generates the final asserted location proof “*ALP*” and sends it back to the *LA*. The location authority validates by verifying the signatures of the asserted location proof using the public key of the witness to ensure it is from the selected witness. Then, the location authority provides the asserted location proof to the prover.
  *ALP = [AReq_LP_, AR, T_ALP_]* (12)
  where *T_ALP_* is the time of asserted location proof creation.
5. The prover does not trust the location authority; therefore, it sends the asserted location proof (provided by location authority) back to the witness for verification to ensure the provided location proof is endorsed by the witness. Therefore, on receiving *ALP*, the prover issues a verification request *VReq* to the witness:
  *VReq = [ALP, T_VPReq_]*. (13)
6. The witness responds the prover by *Yes/No* after validation of *VReq* by sending message “*VR*”
  *V* = [*R, H(ALP), T_V_*] (14a)
  where *R = {Yes*, *No}* and *T_V_* is a time of generation of *V*
  *VR = [V, Sign_Wc_(V)]*. (14b)
7. After successful verification of the asserted location proof, the prover sends the acknowledgement *ACK_ALP_* to the location authority to end the protocol
  *ACK* = [*ALP, VR, ID_DB′i_, T_ACK_*] (15a)
  where *T_ACK_* is the time of acknowledgment generation
  *ACK_ALP_* = [*ACK, Sign_P_(ACK)*]. (15b)
8. After validation of *ACK_ALP_*, the location authority will end the protocol and send *ACK_ALP_′* to *RRSN* after signing it.
  *ACK_ALP_′* = [*ACK_ALP_, Sign_LA_(ACK_ALP_)*] (15c)
9. *RRSN* after validation of *ACK_ALP_′* will make it part of the location provenance chain by broadcasting it in the admin layer.

Once the location proof protocol ends, the prover can present the asserted location proof to any third-party service to avail the location-based services. The third-party can check the validity of the generated location proof by requesting any supervisor node of the admin layer.

## 5. Security Analysis

To highlight the security of MobChain, let us examine the possibilities for fake proof generation by the prover:

1. Suppose all witnesses co-located to the prover are willing to participate in the collusion attack at a particular time instance. Therefore, the probability of getting malicious witnesses selected is 100%. In this scenario, the fake proof generation is only possible if the chosen location authority is also colluding. If the location authority is honest and not willing to collude, then the prover will not be able to get the fake proof of location. However, in the presence of some honest and malicious location authorities, the probability of a successful collusion attack is still reduced in MobChain, as location authority selection control is not in the hands of the prover, which is the weakness of past location proof systems.
2. Suppose all available location authorities are willing to collude with the prover. Therefore, whatever location authority is chosen by the admin layer will allow the prover to obtain a fake proof. However, a fake proof cannot be generated without having a colluding witness. If a chosen witness is honest, then the fake proof will not be asserted by the witness, and the generated proof will be rejected. Since in existing schemes, witness selection control either lies with the location authority or prover itself, therefore, three-way collusion cannot be prevented. However MobChain, due to the separation of participant selection from the location proof protocol, resists such three-way collusion.
3. At least 51% of the supervisor nodes in the admin layer are compromised and are colluding with the prover to assign a malicious witness and location authority of their own choice to enable fake location proof generation. We have assumed that the prover is unable to compromise 51% of the supervisor nodes in the admin layer to get the decision of his own choice [27]. Even if the prover is successful to compromise 51% of the supervisor nodes, without having a colluding location authority and witness, a collusion attack is not possible.
4. All witnesses and location authorities in the system available at a time instance are willing to collude with the prover. It means that the whole location proof system is being compromised by the prover. In this situation, even if every supervisor node in the admin layer is honest, whatever witness and location authority are chosen by the admin layer will allow the prover to generate fake proof successfully following the protocol honestly. However, we assume that at any single instance of time, all the witnesses and location authorities are not willing to collude with the single prover.

It can be deduced from the above discussion that in MobChain, a collusion attack can only be possible if

- All available location authorities and witnesses in the system are malicious and willing to collude with the prover at any time. There is not a single honest location authority and witness available in the system.
- At least 51% of the supervisor nodes in the admin layer are compromised, and the prover makes their own decisions. That is, the colluding location authority and witness are chosen in a decentralized decision.

Furthermore, to elaborate on the possibilities of attacks, we present a security analysis of MobChain by establishing a matrix indicating the status (honest, malicious) of the participants (prover, witness, and *LA*) involved in location proof generation protocol. In Table 2, we represent the participants of the protocol as *Honest* (H) or *Malicious* (M) and list the possible attacks in each case. Moreover, (✓) indicates that scheme can resist attack while (✗) indicates scheme is prone to attack and (NA) means case does not applies to scheme. 

Based on Table 2, we analyze the security of the proposed scheme for each case and explain how it is mitigated.

Case 1—All participants are honest: When all participants are honest, no attack situation exists, the protocol executes normally, and no fake proof generation will be possible.

Case 2—Malicious Witness: Malicious witnesses may try victimizing the honest prover by endorsing different temporal information than the one in the prover request [2,13]. In MobChain, victimization attacks can be detected on two levels. The first false time endorsement by a witness can be detected by the honest location authority and prover by checking *T_AStat_* in Equation (10) from Section 4.3.1 in the assertion response message. The *assertion response (AR)* in Equation (11) is the approval of witnesses about the prover’s location *L_P_* (provided in the proof request in Equation (1)). Secondly, supervisor nodes will be able to detect the false endorsed proof, as the prover’s request time and the assertion time *T_AStat_* of the endorsing witness are not within a certain range (i.e., few milliseconds). Furthermore, decision block information contains the location proof request time, which can also help in validating the spatiotemporal information.

Case 3—Malicious Location Authority: The location authority may try false proof generation for victimizing any prover, but it cannot generate fake proof, as no approval is given by the admin layer. To generate the location proof, the location authority needs to include the *AMsg′* in Equation (6b) to generate the fake proof request. Furthermore, *LA* does not possess the private key for the prover. In addition, *LA* will not receive a final acknowledgement *ACK_ALP_* in Equation (15b) from the prover, and therefore, the proof will not be accepted by supervisor nodes. Another possibility is that *LA* may try to deny the proof request for the prover by deliberately expressing a denial of service attack. With MobChain, since multiple location authorities can be deployed on the same site, therefore, the prover can be assigned an alternate location authority to aid him in a proof generation.

Case 4—Location Authority–Witness Collusion: An implication attack is an example of location authority and witness collusion [3,5]. It is a special case of a location proof system where an innocent prover can be victimized with a false visit claim generated by a colluding location authority and witness. The location authority and witness can collude to generate the fake proof to victimize the prover, but without approval *AMsg′* in Equation (6b) from the admin layer and final acknowledgement *ACK_ALP_* in Equation (15b) from the prover, implication attack is not possible. Reusing or tampering with old *AMsg′* and *ACK_ALP_* can be detected by supervisor nodes because no corresponding decision block will exist in the decision blockchain against the tampered *AMsg′*.

Case 5—Malicious Prover: The prover has full control over his smart device; therefore, they can override the functionality of the mobile application to tamper with the proofs. Attacks possible by a malicious prover include False Presence, False Time, Sequence Alteration, Presence Repudiation, Proof Tampering. In the presence of an honest location authority and witnesses, a prover standalone cannot generate a fake proof. However, since MobChain maintains the decision blockchain and location provenance, any fake proof generated by the malicious prover will be detected and rejected by supervisor nodes. Another possibility is *Prover–Prover Collusion* [9,54], which is also known as *Wormhole/Terrorist Fraud* [9], in which an attack can occur when prover A colludes with prover B who is present at the desired physical location to impersonate as A and generate a location proof for A. A *Terrorist Fraud attack* can easily be detected by honest LA and witnesses due to the delay in responses.

Case 6—Prover–Witness Collusion: A false endorsement [3,5] attack is launched by a colluding witness and prover where the prover does not physically co-locate to the witness, and the witness falsely asserts the location proof to prove the user’s physical presence. In the presence of an honest LA, false endorsement attacks cannot occur [54]. Secondly, witness selection control is not in the hands of the prover, which reduces the probability of such an attack in MobChain.

Case 7—Prover–Location Authority Collusion: A location authority can also be malicious and may collude with the prover. A puppet witness [3] attack is only possible when the prover and location authority collude. The MobChain participant selection mechanism is decentralized, and the prover does not have control over the participant’s choice; therefore, the probability of a puppet witness attack is zero. The witness is chosen by the P2P network from registered witnesses, and *AMsg′* in Equation (6b) contains the selected witness. The location proof generated with a puppet witness attack can be detected, as the witness’s information will not match with the witness’s information in *AMsg′*, as the puppet witness is not registered with the admin layer. Such proof will be discarded by supervisor nodes in the admin layer of MobChain.

Case 8—Prover–Witness–Location Authority Collusion: This scenario is known as *three-way collusion*. To the best of our knowledge, existing schemes have assumed that three-way collusion will not exist. Three-way collusion means the prover, witness, and location authority are all malicious and may be colluding at the same time to generate a false proof of location for the prover. The existing schemes have assumed that all three parties will not be colluding at the same time, which is a strong assumption [13]. However, in actuality, if the prover and location authority are colluding, then certainly, they can involve the colluding witness. For three-way collusion to be successful, *AMsg′* in Equation (6b) is required by the prover, pointing to a malicious *LA* and witness of his own choice. Otherwise, without a valid *AMsg′*, generate proof will be rejected by supervisor nodes. If the prover generates the fake *AMsg′*, then in the final step of location proof generation protocol, it will be detected, as no corresponding decision block will exist in the decision blockchain.

## 6. Experimental Evaluation

For experimental evaluation of the scheme, we have developed a proof-of-concept application [55] to simulate the behavior of MobChain. POC is developed using Java programming language with an AKKA [55] framework, which is an open-source library for the development of scalable distributed applications. The AKKA framework allows the development of reactive actor-based message-driven systems. The actor in the framework is an object that possesses state and behavior. In the case of MobChain, the prover, witness, location authority (LA), and supervisor node (SN) are defined as actors. Each type of actor is capable of communicating with all other actors in the system through messages. Furthermore, contrary to the client-server, each actor in the system is capable of communicating and accepting a connection from other actors in the system. Therefore, the built-in actor system of the AKKA framework enables a peer-to-peer network of actors for MobChain. Moreover, the behavior of each type of actor is mapped to a type of message communicated. In other words, all types of messages received by any actor in the message define corresponding message handlers. At the time of the development of POC, the AKKA framework [55] version 2.11 was used. As mentioned above, the MobChain entities prover, witness, LA, and SN are modeled as actors of the framework. All communication of these actors is based on messages described in schematic description of the distributed consensus and location proof generation protocol in Section 4 of the manuscript.

Simulation results are the average of multiple rounds of proof requests executed on an HP EliteBook with processor Intel (R) Core (TM) i7-3720QM CPU @ 2.60 GHz and 16 GB RAM over Windows 10. We used ECC signatures for the non-repudiation of messages communicated between entities of the protocol. The following properties of the system are considered to analyze the space requirements and performance of the proposed scheme:

- Decentralized Decision Time (DDT): It is the time interval between the location proof request to *RRSN* and the final approval message (created after distributed consensus containing the selected location authority and witness) received by the prover. Measuring the decentralized decision time will help to estimate the additional overhead in proof generation time contributed by distributed consensus.
- Proof Generation Time (PGT): Proof generation time is the interval between the location proof request generated and the final generated proof received by the prover. It should be short enough (within a few seconds) for the system to be practically usable. The proof generation time includes the decentralized decision time.
- Decision Block Size: Decision block size depends on the signature scheme used to provide non-repudiation by all entities involved in the decentralized decision. The size of the decision block will have a direct impact on the overall storage capacity required by the admin layer nodes, as the decision blockchain will be maintained by supervisor nodes.
- Location Proof Size: Since location proofs will be stored on the user’s mobile device, therefore, its size must be appropriate concerning the storage capacity of smart devices. On the other hand, the location proof size also drives the storage capacity of supervisor nodes as the location provenance chain is maintained by the admin layer.

However, the performance of the system and space requirements are directly affected by the following parameters:

1. Number of active workers (active witnesses and *LA*(s));
2. Number of supervisor nodes (in a P2P network of admin layer);
  - Consensus Threshold
3. A key size of a signature scheme.

### 6.1. Impact Analysis of Number of Active Workers

In existing schemes such as WORAL [3,13], all co-located witnesses are registered with the location authority, and no additional computation is required to choose the prover’s co-located witness. Hence, the on-site location authority can quickly compute the co-located witness for the prover. Whereas in MobChain, all location authorities and witnesses are registered in the admin layer of supervisor nodes. MobChain improved the security, as the prover and other participants of the protocol do not know in advance who will participate in the protocol for a certain prover. As a result of the separation of participant selection control from the participant, the possibility of establishing covert channels for the off-site prover to generate fake proof of location becomes impractical. However, on the other hand, the delegation of the control decision introduced the additional overhead of computation for evaluating the co-located workers for the prover. Therefore, on the proof request of the prover, a distributed consensus protocol performs the additional computation to find out the appropriate co-located location authority and witness for the prover. Therefore, the number of active workers (location authorities and witnesses) has an impact on the distributed consensus time and on the overall proof generation time. Furthermore, we have also evaluated the impact of the number of active workers on the decision block size and proof size.

#### 6.1.1. Impact on Decentralized Decision Time and Proof Generation Time

To see the impact of the number of active workers on the decentralized decision time and proof generation time, we have performed tests by keeping 15 supervisor nodes in the admin layer of MobChain and the consensus threshold set to 8. Furthermore, we used the ECC key size of 224 bits for signed messages communication.

**Figure 3:**
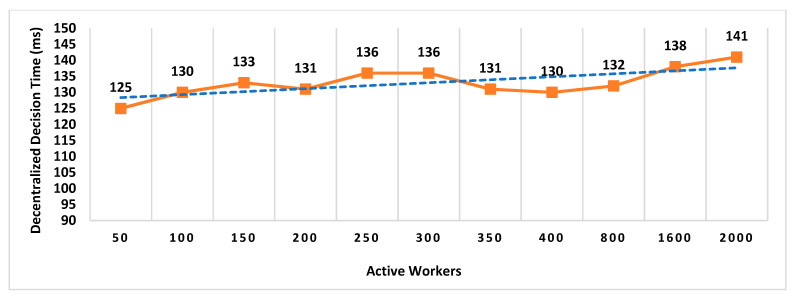
Impact of the number of active workers on the decentralized decision time.

**Figure 4:**
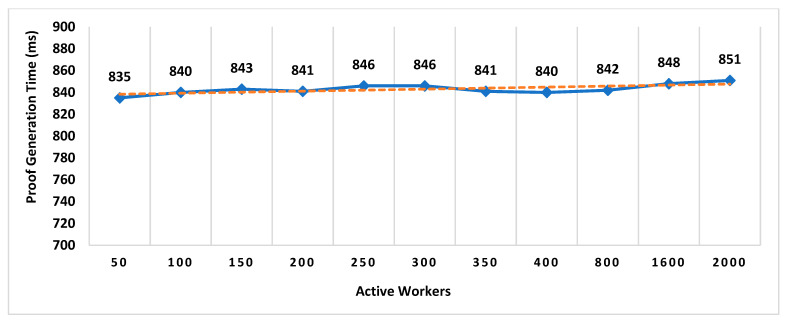
Impact of the number of active workers on the proof generation time.

From Figure 3, a slight increase in the decentralized decision time is observed with an increase in the number of active workers. In situations with a higher worker count, co-located witnesses, and location authorities, the evaluation algorithm might need improvement to reduce this impact of active workers on the decentralized decision time. Especially, the impact of the co-located worker’s selection algorithm will have a higher impact in peak load times where concurrent requests are received by supervisor nodes within a very short interval of milliseconds. Another factor that can impact the overall performance of the decentralized decision time with an increase in the number of active workers is how frequently a worker’s geolocation is refreshed in the admin layer of the P2P network. The specialized cache mechanism can be designed to reduce the time of the co-located worker’s evaluation. However, the cache refreshing mechanism demands control over the frequency of the worker’s geolocation refreshing. The higher the frequency of a worker’s geolocation refreshing, the cache mechanism will start introducing overhead instead of improvement in decentralized decision time. Furthermore, another solution can be the specialized data structure to mitigate the higher frequency of a worker’s geolocation refresh interval while minimizing the co-located workers’ evaluation time. In Figure 4, we have plotted the overall proof generation time against the number of active workers to see what other factors, besides the decentralized decision time, affect the performance of the scheme.

By comparing the graphs in Figure 3 and Figure 4, we can deduce that the proof generation time has very little overhead of decentralized decision. With experimentation, we have identified that much of the proof generation time includes the secure localization time. Secure localization is done twice in the proof generation protocol (i) by the location authority to identify that the prover is physically present in the vicinity, and (ii) by a witness to ensure that the prover is physically co-located. The distance of participants from the Wi-Fi access point has also little impact on secure localization and message communication. In a real scenario, internet bandwidth and factors affecting the communication will affect the decentralized decision time. However, with our POC simulation, we can identify the lower bounds on the performance.

#### 6.1.2. Impact on Proof Size and Decision Block Size

**Figure 5:**
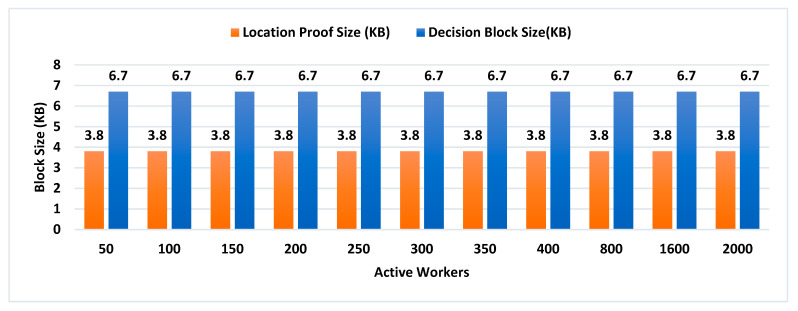
Impact of the number of active workers on the proof size and decision block size.

Location proof size determines the storage requirements from the mobile devices perspective and location provenance chain maintained by the admin layer. The size of the location proof is the primary concern for mobile devices having limited storage capacity. It becomes more of a concern for users when they have to keep the location proofs for a longer period of time. For example, a civil engineer needs to collect location proofs on a daily basis for different sites visited. He needs to keep them and present them to the accounts department at the end of the month. Therefore, a larger size of location proofs will make the scheme impractical. Therefore, we measured the size of the location proof generated and the decision block generated by RRSN in the location proof generation process. By flooring the value of size in KB, we plotted the graph in Figure 5. Based on the results, we can deduce that the number of active workers does not affect the size of the location proof and decision block. The location proof protocol involves only three participants (prover, location authority, and witness); therefore, the overall number of active workers in the system does not impact the size of the location proof. In the same way, the decentralized decision is dependent on the consensus threshold (i.e., 51% of supervisor nodes agreeing on the same location authority and witness selection); therefore, the decision block size will remain within a certain size range as long as a total number of supervisor nodes in the system remain constant. Therefore, we can conclude that the storage space requirements are dependent on the size of the admin layer, while it is independent of the size of the service layer of MobChain. Furthermore, an upper bound of decision block size can be used to determine the storage capacity requirement for the admin layer.

### 6.2. Number of Supervisor Nodes Impact Analysis

As discussed earlier, in MobChain, the decentralized decision for the selection of participants is introduced as an additional step in location proof protocol. The decentralized decision is taken by supervisor nodes in the admin layer, and therefore, the size of this P2P network has an impact on the decision time and decision block size. However, practically, the consensus threshold is the key factor, which controls the impact of how many supervisor nodes determines the reliability of the decision. To measure the impact of the consensus threshold, we performed the experimentation, keeping the number of supervisor nodes constant i.e., 15, and active workers i.e., 400 with an ECC key size of 224 bits.

#### 6.2.1. Impact on Decentralized Decision Time and Proof Generation Time

**Figure 6:**
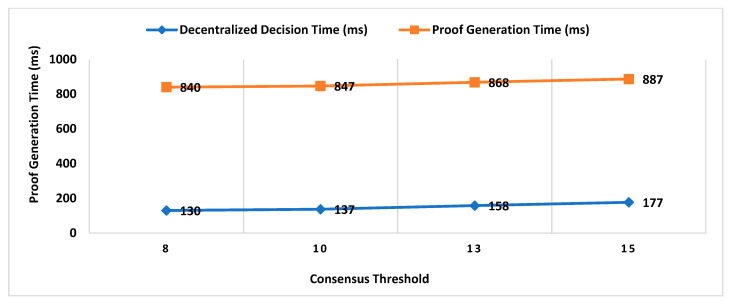
Consensus threshold impact on the decentralized decision time and proof generation time.

In Figure 6, we have measured the impact of the consensus threshold increase on decentralized decision time and overall proof generation time. As the location proof protocol cannot start until the decentralized decision for participants selection completes, therefore, the proof generation time includes the decentralized decision time. Furthermore, in distributed consensus explanation, the decentralized decision time is controlled by a consensus threshold value (*N*/2) + *K*, which determines that how many supervisor nodes need to agree on the same participants to establish the decision. Therefore, in our experimentation, to evaluate the impact of the threshold value, we gradually increased the value of *K* and plotted the graph of the results. We observed a slight increase in the decentralized decision time with an increase in the consensus threshold value.

It means that a higher number of supervisor nodes and a higher value of the consensus threshold can increase the decentralized decision time and overall proof generation time. Additionally, the communication channel between the supervisor nodes is another factor that will enhance the impact on decentralized decision time with an increase in the size of the admin layer network. Furthermore, this impact is also affected by another factor; i.e., ECC key size. We repeated the same experiment by increasing the key size of the ECC signature scheme.

**Figure 7:**
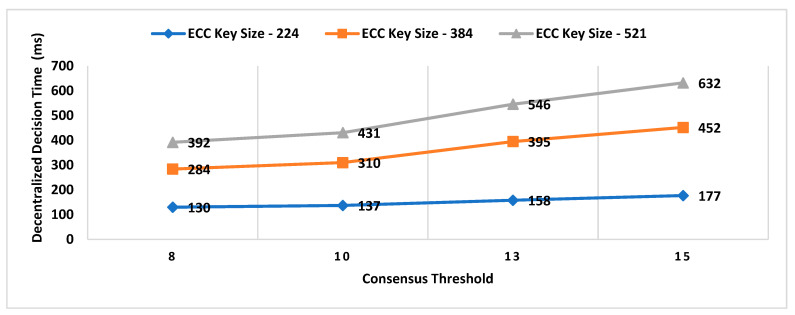
Consensus threshold and key size impact on decentralized decision time.

**Figure 8:**
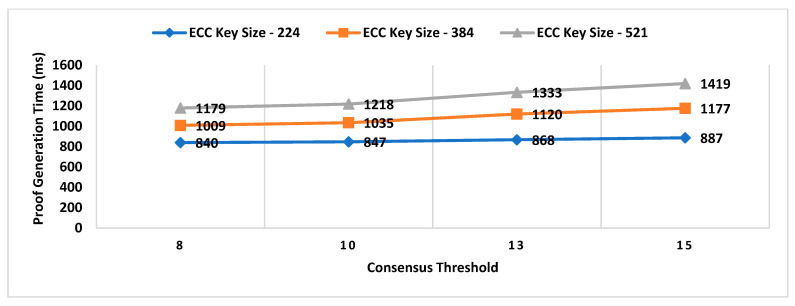
Consensus threshold and key size impact on proof generation time.

From Figure 7 and Figure 8, we can observe the impact of the key size on the decentralized decision time and overall proof generation time. Based on the results, we can deduce that the total number of supervisor nodes, the value of the consensus threshold, and the signature scheme’s key size collectively determine the decentralized decision time. However, the key size also has an impact on the performance of the location proof generation phase, as all messages between the prover, location authority, and witness are signed and validated during location proof generation protocol. The larger the key size, the more the messaging signing time increases with the increase of the decision time and proof generation time.

#### 6.2.2. Impact on Location Proof Size and Decision Block Size

**Figure 9:**
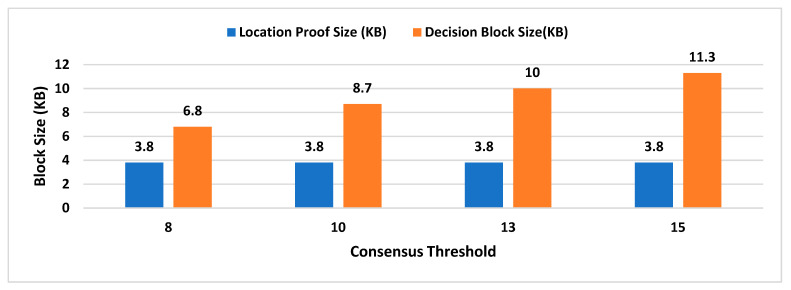
Consensus threshold impact on location proof size and decision block size.

**Figure 10:**
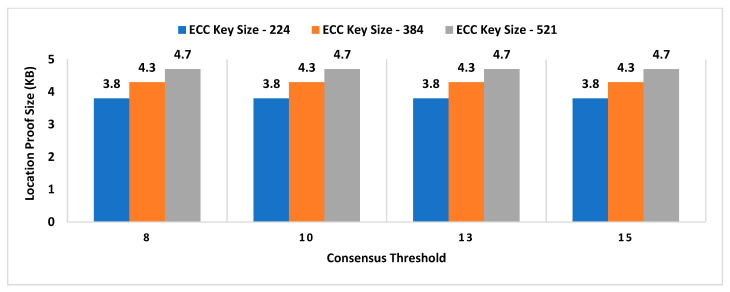
Consensus threshold and key size impact on location proof size.

From the results in Figure 9, we can deduce that the location proof size is independent of the consensus threshold, while the decision block size is increased with an increase in consensus threshold. The location proof size and decision block are independent of each other. The location proof only contains the decision block ID, which is a few bytes. However, the reason for the increase in the decision block size is the inclusion of many confirmations of supervisor nodes with the information of chosen workers. As soon as 51% of the supervisor nodes develop consensus on the same set of workers for aiding the prover in a proof generation, confirmations received by RRSN up to this time are made part of the decision block. Therefore, in the presence of malicious or malfunctioning or outdated supervisor nodes, reaching a consensus can vary, and it may cause an increase in the size of the decision block. With the help of these confirmations, a decision block is validated by all supervisor nodes before making it part of the decision blockchain on the completion of the protocol. Secondly, the decision block can be validated later to detect any tampering by a malicious supervisor node or any corruption. Furthermore, we evaluated the impact of key size along with a consensus threshold on the location proof size and decision block size. Figure 10 shows the results of the location proof size against each key size and consensus threshold value. Keeping the number of supervisor nodes constant; i.e.,15, some active workers i.e., 400 and an ECC key size of 224 bits, the results of the consensus threshold impact on the location proof size and decision block size are plotted in Figure 10.

**Figure 11:**
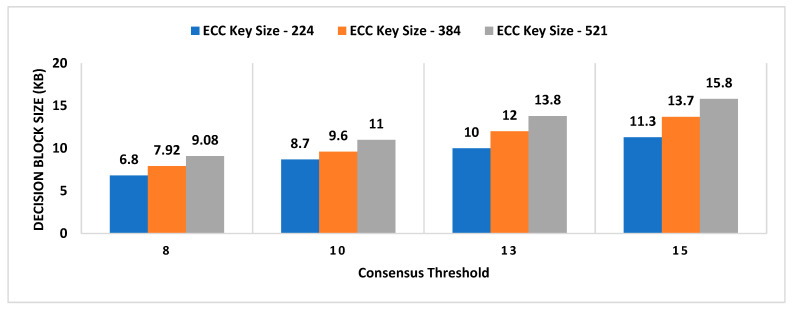
Consensus threshold and key size impact on decision block size.

From the results, it is obvious that the location proof size is independent of the consensus threshold; however, the key size increase directly increased the proof size. The reason is the signed information communicated between participants of the protocol during location proof generation. We can deduce the upper and lower bound on the size of the proof from Figure 10 and estimate the storage requirements for smart devices. However, the key size and consensus threshold impact will be higher on the decision block size. We can compare the results of Figure 9 and Figure 11 to see the difference of consensus threshold standalone impact on decision block size and with an increase in key size, respectively. An almost 1 KB increase in size is introduced in the decision block with an increase in key size. The reason is that with an increase in key size, the signature size included in each confirmation message increases; therefore, it increases the overall size of the decision block. Furthermore, the higher the consensus threshold value, the greater the number of signed confirmations that are included in the decision block resulting from an increase in size.

### 6.3. Concurrent Request Impact on Decentralized Decision Time

**Figure 12:**
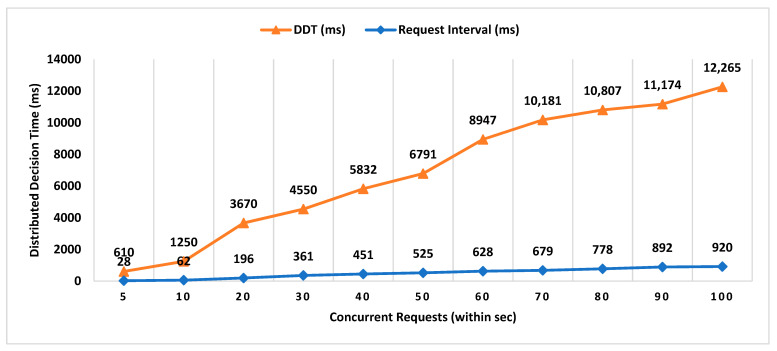
Concurrent request impact on decentralized decision time.

Another important factor determining the performance of the MobChain is the number of concurrent requests to supervisor nodes by provers. To see the impact of concurrent requests on performance when all requests are received by the same supervisor node, we performed the tests by keeping the number of supervisor nodes in the admin layer constant; i.e., 15; the number of active workers is 1600, and the consensus threshold is 8 with an ECC key size of 224. The results of the experiment are plotted in Figure 12.

All concurrent requests are generated in less than 1 s on the same supervisor node to see the impact on decentralized decision time. In Figure 12, the *“Request Interval”* is the duration within which all requests are received by RRSN. For example, five concurrent requests are received by RRSN within 28 milliseconds, and 100 concurrent requests are received within 920 milliseconds. Against each request, RRSN performs the following tasks: (i) broadcasts the location proof request to the admin layer to initiate the distributed consensus against each request; (ii) computes the co-located workers for the prover; (iii) validates the confirmations received against each proof and waits until the consensus threshold is achieved against each request; (iv) generates a decision block once consensus is established on the same workers for the prover against an individual request; (v) broadcasts the decision block to the admin layer to make it part of a decision blockchain; (vi) sends the approval message, including the decision block ID against the individual proof request, to the prover, selected location authority, and witness. Therefore, Figure 12 can give an insight into the impact of concurrent requests load on a single supervisor node, and we can see the degradation of performance from results. Hence, we need to re-evaluate the protocol to identify the improvements for reducing the impact of concurrent requests within a short interval on a single supervisor node.

In the light of all the experimentation, we can review the capabilities of MobChain in comparison with the WORAL and STAMP schemes in Table 3.

Based on the results, the optimal parameter configurations for MobChain are

- ECC key size 224;
- Consensus threshold (*N*/2) + 1;
- 15 supervisor nodes.

However, the number of concurrent requests for the same supervisor nodes is a critical area that needs improvement. Whereas, for a low load of concurrent requests, the MobChain performance is close to WORAL while providing the resistance to three-way collusion. With optimal parameter configurations, MobChain can compete for WORAL in performance; however, the storage requirements of MobChain are high concerning both smart devices and commodity machines used as supervisor nodes. Furthermore, the ECC key size of 224 bits provides the same level of security as that of RSA with a key size of 2048 bits, and an ECC key size of 521 bits is equivalent to an RSA key size of 15,360 bits. Since the WORAL proof generation time is ≤1 sec with an RSA key size 2048 bits, MobChain with a consensus threshold of 8 and ECC key size of 512 bits has matching performance with stronger security.

## 7. Conclusions and Future Work

Witness-oriented location proof protocols with location provenance paved the path for improving real-life business operations. However, the trustworthiness of location proofs is a real concern, as participants of the protocol may collude.

All participants of witness-oriented location proof can be malicious and colluding at the same time, which enables the three-way collusion attacks and allows fake proof generation. Therefore, trustworthiness cannot be guaranteed without resistance to three-way collusion. All existing schemes are unable to resist three-way collusion due to the in-built problem of having participant selection control in the hands of one of the participants. Secondly, participant selection control lies with a single party, which makes collusion attacks possible. MobChain resolved these design weaknesses by first removing the control decisions from participants of the location proof protocol, secondly making these control decisions decentralized. Therefore, MobChain introduced the distributed consensus for participant’s selection in location proof generation protocol of witness-oriented schemes to provide resistance against three-way collusion. Furthermore, the decentralized centralized decisions introduce the overhead of an additional step in the location proof system, and the MobChain proof-of-concept application provides the lower and upper bounds on the performance and storage requirements. Comparisons with the state-of-the-art solutions show that MobChain is computationally efficient and highly available while improving the security of LPS.

In the future, we intend to further optimize the algorithm of witness and location authority selection to reduce the impact of the number of active workers registered on the MobChain network. With the increase in the number of location authorities and witnesses, decentralized decision time (DDT) becomes crucial as it impacts the proof generation time. The number of active workers (location authorities and witnesses) in the system and the concurrent number of proof requests are the two factors decisive for the performance of MobChain decentralized decision time. For each prover’s request, all active workers are evaluated for the identification of co-located entities in the decentralized decision. Therefore, in the case of thousands of workers connected to the peer-to-peer network of MobChain may result in increased decentralized decision time, making it impractical. Initial experimentation has shown that a higher number of concurrent requests has a drastic impact on the decentralized decision time. To deal with the performance issue with a higher number of active workers and a concurrent number of proof requests for supervisor node, a specialized data structure or an algorithm is required, which can filter out co-located entities from a big set of available workers in an efficient manner. Furthermore, location proof size and decision block size need to be optimized to reduce the storage requirements, especially for mobile devices. Moreover, we need to transform the proof-of-concept application to a production-ready application to test it in a real environment.

## Figures and Tables

**Table 1:** Abbreviations and their description.

Abbreviation	Term	Description
*LA*	Location Authority	The designated stationary entity on each site aids the prover in location proof generation while ensuring that the witness and prover are physically present at the location.
*WN*	Worker Node	Worker node refers to LA(s) and witnesses who provide services to prover for the generation of the asserted location proof.
*SN*	Supervisor Node	Admin layer peers are called supervisor nodes. These nodes are responsible for decentralized decisions.
*RRSN*	Request-Receiving Supervisor Node	The supervisor node (*SN*) initiates the distributed consensus protocol on receiving the proof request from the prover. Any of the supervisor nodes can receive the location proof request. *RRSN* is a label to differentiate the role of the request-receiving supervisor node from other supervisor nodes in the system. For example, Prover1 requests SN1 for location proof; then, SN1 is considered *RRSN*, while at that same time, Prover2 requests SN2 for location proof; then, SN2 is considered RRSN in for the Prover2 request.
*CWN*	Chosen Worker Node	*CWN* refers to the *LA* and witness chosen by the admin layer through distributed consensus against the proof request of the prover.
*PReq*	Proof Request	Proof request message symbol.
*DAM*	Decision Acknowledgement Message	During consensus protocol execution, every *SN* decides the witnesses and *LA* for the prover and informs the *RRSN* about his choice by sending a decision acknowledgement message.
*DB*	Decision Block	The decision block is the final message generated by *RRSN* on the completion of distributed consensus. This decision block is made part of the decision blockchain.
*AMsg*	Approval Message	On completion of distribution consensus, the prover is provided with the approval message containing the decision block ID, chosen witness, and location authority.
*LPReq*	Location Proof Request	The prover requests the location authority to aid him in proof generation using an approval message as an authentication token.
*LP*	Location Proof	*LP* is a digital certificate generated by *LA* approving the physical presence of the prover at the location on a specific time instance.
*AReq_LP_*	Assertion Request (of Location Proof)	The location authority requests the witness for *LP* assertion by sending *AReqLP.*
*AR*	Assertion Response	Witness after successful verification of the prover’s location and validation of *AReqLP* sends back an assertion response (*AR*) to the location authority.
*ALP*	Asserted Location Proof	Witness-asserted *LP* is termed as *ALP*.
*VReq*	Verification Request	To ensure the validity of *ALP*, the prover issues a verification request *VReq* to witness.
*VR*	Verification Response	Witness responds to the prover by Yes/No after the validation of *VReq* by sending the message “*VR*”
*ACK_ALP_*	Acknowledgement Message	On successful verification of the asserted location proof, the prover sends the acknowledgement *ACK_ALP_* to the location authority to end the protocol.

**Table 2:** Malicious participants and possible collusion attacks.

Case	Prover	LA	Witness	Scenario/Collusion Class	Threat/Attack	STAMP [9]	WORAL [13]	MobChain
1	H	H	H	Everyone Honest	No attack	✓	✓	✓
2	H	H	M	Malicious Witness	False endorsement	✓	✓	✓
3	H	M	H	Malicious LA	Denial of service, False assertion	NA	✓	✓
4	H	M	M	LA–Witness Collusion	Implication attack	NA	✓	✓
5	M	H	H	Malicious Prover, Prover–Prover Collusion	False presence, Proof tampering, Sequence alteration, False time, Wormhole/Terrorist fraud	✓	✓	✓
6	M	H	M	Prover–Witness Collusion	False endorsement	✓	✓	✓
7	M	M	H	Prover–LA Collusion	Puppet witness attack	NA	✓	✓
8	M	M	M	Three-Way Collusion	Fake proof generation is achievable when all participants are malicious at the same time.	✗	✗	✓

**Table 3:** Comparison with existing techniques.

Feature	STAMP [9]	WORAL [13]	MobChain
Proof Generation Time (s)	≤3	≤1	≤1
Proof Size (KB)	≤1.3	≤2	≥4.7
Number of Entities Involved in the Protocol	Multiple	3	3
Malicious LA	Partial	Yes	Yes
Vulnerability (%)	≥75	12.5	0
Decentralized Decision Time (ms)	NA	NA	≤200
Decision Block Size (KB)	NA	NA	≥6.8

## Data Availability

Not applicable.
